# Carotid Intima-Media Thickness and Its Correlation With Echocardiographic Left Ventricular Function and Geometry in Hypertensive Individuals: A Cross-Sectional Study

**DOI:** 10.7759/cureus.47589

**Published:** 2023-10-24

**Authors:** Mariam Inuwa, Janet N Ajuluchukwu, Akinsanya Olusegun-Joseph

**Affiliations:** 1 Internal Medicine, Lagos University Teaching Hospital, Lagos, NGA

**Keywords:** echocardiography, diastolic function, left ventricular geometry, atherosclerosis, carotid intima-media thickness (cimt), cardiovascular diseases, cross-sectional study

## Abstract

Background

It is important to consider left ventricular hypertrophy (LVH) and carotid intima-media thickness (CIMT) in assessing hypertensive patients’ global cardiovascular risk profile, as LVH and arterial wall changes occur concurrently. This study aimed to assess the relationship between CIMT and left ventricular geometry and function in hypertensive patients.

Methodology

This cross-sectional study included 200 hypertensive individuals and sought to correlate their CIMT with left ventricular geometry and function in Lagos University Teaching Hospital. Hypertension was defined as blood pressure ≥140/90 mmHg or on treatment for hypertension presenting at the outpatient clinics. Patients who satisfied the inclusion criteria were recruited. Abnormal CIMT was defined as >0.9 mm. Patients’ demographic data were obtained in addition to general characteristics, physical examination, transthoracic echocardiography, and CIMT. The statistical relationship between CIMT and left ventricular geometry and function was obtained and analyzed.

Results

Normal geometry and LVH were observed in 50.5% and 15.5%, respectively. Left ventricular geometry was associated with abnormal CIMT (χ^2^ = 31.688, p < 0.001). Furthermore, the mean left ventricular mass index was statistically different between abnormal and normal CIMT (97.84 ± 30.5 vs. 80.75 ± 15.6; p < 0.001). Regarding left ventricular function, there was no significant difference in E-point septal separation, left ventricular fractional shortening, and left ventricular ejection fraction in abnormal versus normal CIMT groups. However, there was a significant association of CIMT with grades of diastolic dysfunction (χ^2^ = 7.069, p = 0.029). Additionally, individual parameters of diastolic dysfunction such as left atrial volume index and septal mitral were significantly different (p < 0.001).

Conclusions

There was an association between age, left ventricular geometry, diastolic function, and CIMT in hypertensive individuals. Therefore, it is beneficial to evaluate CIMT and for these patients to receive more targeted blood pressure control which may reduce the risk of cardiovascular diseases.

## Introduction

Systemic hypertension is the most common cause of cardiovascular disease which is the leading cause of mortality worldwide [1]. The prevalence of hypertension globally was estimated to be 1.28 billion adults aged 30-79 years [2]. Left ventricular hypertrophy (LVH) is an important marker of cardiovascular risk among hypertensive subjects [3]. LVH is also associated with increased carotid intima-media thickness (CIMT), and hypertension is said to have the greatest effect on CIMT through medial hypertrophy [4]. Increased knowledge on the global cardiovascular risk profile of hypertensives including CIMT would help reduce morbidity and mortality.

Cardiovascular diseases (CVDs) are a group of disorders of the heart and blood vessels which include coronary heart disease, heart failure, hypertension, cerebrovascular disease, and peripheral artery disease [5,6]. The screening tools for CVDs include blood pressure measurement, dyslipidemia, C-reactive protein, coronary artery calcium score, abdominal aorta ultrasound, electrocardiography, genetic screening, and carotid artery ultrasound, which is used as a marker of atherosclerosis [7]. One such method for predicting organ damage is the measurement of CIMT [8]. LVH and thickened CIMT are well-documented sequelae of long-standing hypertension [9].

LVH is an increase in left ventricular (LV) mass and a complication of hypertension [10,11]. LVH is an adaptive response to chronic pressure overload [11]. LVH can be classified as concentric, eccentric, and concentric LV remodelling [12]. Concentric LVH is defined as increased relative wall thickness with increased LV mass [12]. When the relative wall thickness is normal with increased LV mass, LVH is classified as eccentric [12]. Concentric LV remodelling occurs when relative wall thickness increases with normal LV mass [12]. In LVH, there is an increase in the size of the cardiomyocyte with increased fibrosis and abnormalities of the intramyocardial coronary vasculature [13,14]. LVH and abnormal LV geometry are both important markers of cardiovascular risk among hypertensive subjects [3], and the risk increases with concentric LVH [10]. The Framingham Study has shown that increased LV mass is an independent predictor of cardiovascular events [15]. Complications of LVH include atrial fibrillation, heart failure, and sudden death [11]. Early recognition and improved understanding of cardiac hypertrophy lead to better therapeutic strategies for this cardiovascular risk factor [11].

Increased CIMT is highly associated with atherosclerosis and may be regarded as a marker of generalized subclinical atherosclerosis [16,17]. Measurement of CIMT through ultrasound has become a valuable tool for detecting and monitoring the progression of atherosclerosis [18]. CIMT is measured using B-mode ultrasonography and Doppler (duplex), which is non-invasive, safe, and reproducible [18]. It is accepted as one of the best methods for the evaluation of arterial wall structure [18]. Hypertension is said to have the greatest effect on intima-media thickness (IMT) through medial hypertrophy, a process specifically related to the disease [4].

CIMT is not routinely assessed but plays an important role in cardiovascular risk stratification [19]. Studies in Nigeria have assessed CIMT in hypertensives, diabetics, and renal disease but there is a dearth of data on CIMT assessment and correlation with LV geometry and function. Therefore, assessment of CIMT as a non-invasive measure of CVD burden in adults [20] with early identification is beneficial [21]. This study aims to assess the relationship between CIMT and LV geometry and function in hypertensives. This will further impact the risk stratification of hypertensives.

## Materials and methods

This was a cross-sectional study conducted among hypertensive individuals. The participants were recruited from Lagos State University Teaching Hospital in the outpatient clinic of the cardiology department. The study was conducted for six months. A total of 200 participants were recruited in the study to achieve an estimated sample size using Fisher’s formula [22]. A convenient sampling method was used to select the individuals that constituted the study group.

Ethical approval was obtained from the Lagos State University Teaching Hospital Health Research Ethics Committee) (approval number: ADM/DCST/HREC/APP/2668).

A structured questionnaire that contained the patient’s demographics, clinical variables, and the current antihypertensive therapy was administered to each patient. Physical examination, carotid ultrasound, and echocardiography were also conducted for each patient. Investigations done in the last three to six months needed to profile patients were extracted from case notes to aid inclusion and exclusion. We included patients who provided informed consent, were aged ≥18 years, and had a confirmed diagnosis of hypertension. We excluded patients aged <18 years; patients with diabetes mellitus; smokers; those with a previous history of coronary artery disease, chronic kidney disease (estimated glomerular filtration rate <60 mL/minute), stroke, or transient ischemic attack; pregnant women; those with high carotid artery bifurcation; and those with a short neck. These exclusion criteria have been shown to have increased CIMT and/or LVH [8,23-26].

The left ventricular ejection fraction (LVEF) was calculated automatically by the machine using the Teicholz method [27]. LV diastolic function was assessed according to the guidelines of the 2016 American Society of Echocardiography recommendations for the evaluation of LV diastolic dysfunction [28]. LV diastolic function was assessed using transmitral Doppler velocities and tissue Doppler of the septal and lateral mitral annulus [28].

Carotid ultrasound was conducted bilaterally on each patient, and IMT values of more than 0.9 mm (ESC) were considered abnormal [16].

Data were imputed and analyzed using SPSS version 25.0 (IBM Corp., Armonk, NY, USA). Categorical data were presented as frequencies, percentages, or proportions, while continuous data were presented as means and standard deviation or median and interquartile range when skewed. The Kolmogorov-Smirnov test was used to assess normal distribution.

Mean comparisons between two groups were performed by independent Student’s t-test for continuous variables, while the association between CIMT categories and other categorical variables was assessed using the chi-square and Fisher exact test. The Pearson correlation coefficient was used to assess the linear relationship between numeric variables. A p-value of less than 0.05 was considered statistically significant at a 95% confidence interval. Charts were used for data presentation where appropriate.

## Results

Demographic characteristics of hypertensives

A total of 200 participants were recruited in this study. Overall, 155 (77.5%) had normal CIMT and 45 (22.5%) had abnormal CIMT. Increasing age was strongly associated with abnormal CIMT (χ^2^ = 11.386, p = 0.023); thus, the frequency of abnormal CIMT was 2 (7.7%) in those aged <40 years and 4 (15.4%) in those aged >70 years. There was no significant difference in mean CIMT between male and female hypertensive as well as their body mass index (BMI) status. However, the prevalence of abnormal CIMT was higher in males (20, 25.6%) than in females (25, 20.5%) but did not achieve statistical significance (p = 0.395). Moreover, the prevalence of abnormal CIMT was the highest in obese patients but did not attain statistical significance (p = 0.245). Similarly, hypertension duration and hypertension control status were also significantly associated with abnormal CIMT (p = 0.018 and p = 0.002, respectively) (Table 1).

**Table 1:** Demographic characteristics of patients. *: P-value <0.05 is statistically significant. BMI = body mass index; CIMT = carotid intima-media thickness

	Abnormal CIMT (n = 45)	Normal CIMT (n = 155)	χ^2^	P-value
	N (%)	N (%)		
Age group (years)			11.386**	0.023*
≤40	2 (7.7)	24 (92.3)		
41–50	5 (10.6)	42 (89.4)		
51–60	20 (29.9)	47 (70.1)		
61–70	14 (31.8)	30 (6.2)		
>70	4 (25.0)	12 (75.0)		
Gender			0.723	0.395
Male	20 (25.6)	58 (74.4)		
Female	25 (20.5)	97 (79.5)		
BMI status			4.157**	0.245
Underweight	1 (50.0)	1 (50.0)		
Normal	8 (17.8)	32 (82.2)		
Overweight	13 (17.8)	69 (82.0)		
Obese	23 (28.7)	57 (71.3)		
Duration since diagnosis of hypertension (years)			11.899	0.018*
≤5	23 (25.6)	67 (74.4)		
6–10	5 (8.2)	56 (91.8)		
11–15	5 (33.3)	10 (66.7)		
16–20	7 (36.8)	12 (63.2)		
>20	5 (33.3)	10 (66.7)		
Hypertensive control status			9.162	0.002*
Uncontrolled	27 (33.3)	54 (66.7)		
Controlled	18 (15.1)	101 (84.9)		

LV geometry in hypertensives

**Figure 1:**
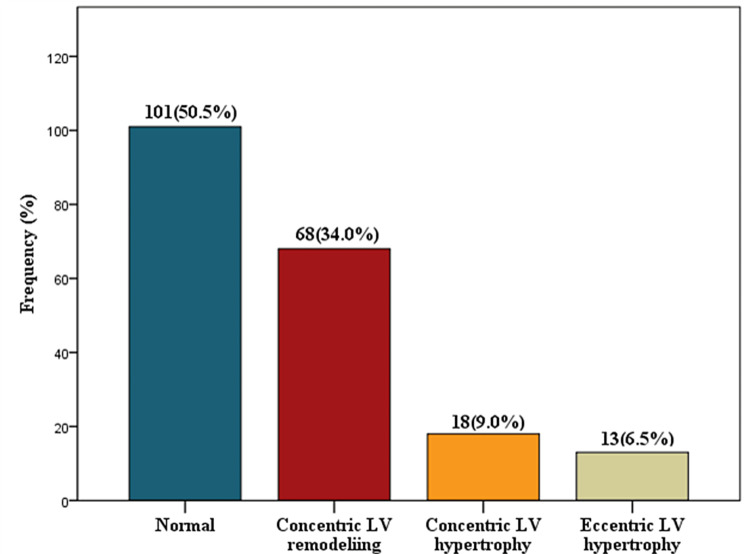
LV geometry among hypertensives. LV = left ventricular

The histogram showed the distribution of the various types of geometry identified in the study. Overall, 101 (50.5%) participants had normal geometry, whereas others had the following abnormal geometries: LV remodeling, LV concentric hypertrophy, and LV eccentric hypertrophy at 68 (34%), 18 (9%), and 13 (6.5%), respectively (Figure 1).

Echocardiographic parameters among hypertensive participants

The mean systolic function parameters of E-point septal separation (EPSS) of 0.39 ± 0.1 cm, fractional shortening of 39.64 ± 5.1%, and ejection fraction of 70.23 ± 6.2% were within normal limits. No statistically significant difference was found between males and females (p > 0.05). The calculated mean left atrial volume index (LAVI) of 30.35 ± 8.0 mL/m^2^, mitral E/A of 1.01 ± 0.3, septal mitral e of 8.42 ± 2.2 cm/second, and mitral E/e of 8.46 ± 1.9 showed no statistical significance between males and females (p > 0.05), as summarized in Table 2.

**Table 2:** Echocardiographic parameters among hypertensives. *: P-value <0.05 is statistically significant. LVEDD = left ventricular end-diastolic diameter; LVSD = left ventricular end-systolic diameter; LVPWD = left ventricular posterior wall thickness in diastole; EPSS = E-point septal separation; EF = ejection fraction; FS = fractional shortening; LVMI = left ventricular mass index; LAVI = left atrial volume index; IVSD = interventricular septal wall thickness in diastole

	Overall, mean ± SD	Male (n = 78), mean ± SD	Female (n = 122), mean ± SD	t-value	P-value
Left atrium	3.43 ± 0.4	3.47 ± 0.4	3.40 ± 0.4	1.049	0.295
LVEDD	4.56 ± 0.5	4.75 ± 0.6	4.44 ± 0.5	4.262	<0.001*
LVESD	2.74 ± 0.5	2.87 ± 0.5	2.65 ± 0.4	3.684	<0.001*
IVSD	1.02 ± 0.1	1.07 ± 0.2	1.00 ± 0.1	3.152	0.002*
LVPWD	0.95 ± 0.1	0.98 ± 0.1	0.92 ± 0.1	3.448	0.001*
EPSS	0.39 ± 0.1	0.43 ± 0.1	0.36 ± 0.1	1.833	0.068
LV FS	39.64 ± 5.1	39.12 ± 4.9	39.98 ± 5.1	-1.176	0.241
LV EF	70.23 ± 6.2	69.38 ± 6.4	70.76 ± 6.1	-1.532	0.127
LV mass	156.60 ± 47.1	176.73 ± 57.8	143.72 ± 33.2	5.129	<0.001*
LVMI	39.64 ± 5.0	90.53 ± 25.0	80.81 ± 17.3	3.249	0.001*
LA volume	55.73 ± 15.3	58.40 ± 17.5	54.02 ± 13.5	1.987	0.048*
LAVI	30.35 ± 8.0	30.13 ± 8.0	30.49 ± 8.1	-0.307	0.759
Mitral E	69.89 ± 17.8	68.20 ± 17.3	70.96 ± 18.1	-1.075	0.284
Mitral A	72.96 ± 16.4	73.48 ± 16.3	72.64 ± 16.5	0.354	0.723
Mitral E/A	1.01 ± 0.3	0.98 ± 0.3	1.03 ± 0.4	-0.995	0.321
Deceleration time	0.22 ± 0.04	0.22 ± 0.05	0.23 ± 0.04	-2.018	0.045*
Septal mitral e	8.42 ± 2.2	8.29 ± 2.1	8.50 ± 2.3	-0.655	0.513
Septal mitral a	9.22 ± 1.9	9.97 ± 2.0	8.74 ± 1.6	4.811	<0.001*
Mitral E/e	8.46 ± 1.9	8.45 ± 2.0	8.47 ± 1.9	-0.072	0.943

LV geometry and CIMT in hypertensives

Abnormal CIMT occurred only in 45 (15.8%) hypertensive participants with normal LV geometry, with abnormal CIMT occurring mainly in participants with eccentric LVH and concentric LVH. Overall, 68 had concentric LV remodeling, with 58 (85.3%) having normal CIMT. There were 13 hypertensives with eccentric LV hypertrophy and 18 hypertensives with concentric LV hypertrophy, of whom 5 (38.5%) and 7 (38.9%) had normal CIMT, respectively, as illustrated in Table 3.

**Table 3:** Chi-square analysis of CIMT and LV geometry among hypertensives. *: P-value <0.05 is statistically significant. CIMT = carotid intima-media thickness; LV = left ventricular

	Abnormal CIMT (n = 45)	Normal CIMT (n = 155)	χ^2^	P-value
	N (%)	N (%)		
Left ventricular geometry			31.688	<0.001*
Normal	16 (15.8)	85 (84.5)		
Concentric LV remodeling	10 (14.7)	58 (85.3)		
Eccentric LV hypertrophy	8 (61.5)	5 (38.5)		
Concentric LV hypertrophy	11 (61.1)	7 (38.9)		

LV diastolic function of hypertensives with normal and abnormal CIMT

Table 4 summarizes the diastolic function of hypertensive participants in relation to CIMT. Abnormal CIMT was seen in 15.7% of hypertensive participants with normal diastolic function but was much more seen in grade II diastolic dysfunction where half of the participants (50%) had abnormal CIMT. Other mean diastolic parameters with abnormal CIMT were LAVI at 34.27 ± 11.8 mL/m^2^, mitral E/A at 0.97 ± 0.4, septal mitral e at 7.84 ± 1.8 cm/second, and mitral E/e at 8.56 ± 2.2 (Table 5). There was a significant association between diastolic function and CIMT (χ^2^ = 7.069, p = 0.029) (Table 4). Similarly, some diastolic parameters were statistically significant, including LAVI (p < 0.001) and septal mitral e (p = 0.044), while others showed no significant difference, such as mitral E/e (p = 0.697) and mitral E/A (p = 0.421) (Table 5).

**Table 4:** Chi-square analysis of diastolic function and CIMT in hypertensives. *: P-value <0.05 is statistically significant; **: Fisher’s exact test. CIMT = carotid intima-media thickness

	Abnormal CIMT (n = 45)	Normal CIMT (n = 155)	Total	χ^2^	P-value
	N(%)	N(%)			
Left ventricular diastolic function				7.069**	0.029*
Normal	16 (15.7)	86 (84.3)	102 (100.0)		
Grade I dysfunction	28 (28.3)	66 (71.7)	92 (100.0)		
Grade II dysfunction	3 (50.0)	3 (50.0)	6 (100.0)		

**Table 5:** Comparison of echocardiographic parameters with CIMT in hypertensives. *: P-value <0.05 is statistically significant CIMT = carotid intima-media thickness; LVEDD = left ventricular end-diastolic diameter; LVSD = left ventricular end-systolic diameter; LVPWD = left ventricular posterior wall thickness in diastole; EPSS = E-point septal separation; EF = ejection fraction; FS = fractional shortening; LVMI = left ventricular mass index; LAVI = left atrial volume index; IVSD = interventricular septal wall thickness in diastole

	Abnormal CIMT (n = 45), mean ± SD	Normal CIMT (n = 155), mean ± SD	t-value	P-value
Left atrium	3.64 ± 0.5	3.36 ± 0.4	3.956	<0.001*
LVEDD	4.69 ± 0.7	4.53 ± 0.5	1.882	0.061
LVESD	2.81 ± 0.6	2.72 ± 0.4	1.134	0.258
IVSD	1.11 ± 0.2	1.00 ± 0.1	4.886	<0.001*
LVPWD	1.02 ± 0.2	0.92 ± 0.1	4.638	<0.001*
EPSS	0.43 ± 0.1	0.38 ± 0.1	0.967	0.334
LV EF	70.31 ± 7.3	70.20 ± 5.9	0.105	0.916
LV FS	40.02 ± 5.6	39.53 ± 4.9	0.576	0.565
LV mass	185.33 ± 72.8	148.3 ± 32.3	4.909	<0.001*
LVMI	97.84 ± 30.5	80.75 ± 15.6	5.058	<0.001*
LA volume	65.84 ± 23.0	52.79 ± 10.6	5.373	<0.001*
LAVI	34.27 ± 11.8	28.92 ± 5.9	4.930	<0.001*
Mitral E	68.03 ± 21.9	70.43 ± 16.4	-0.796	0.427
Mitral A	73.35 ± 16.5	72.85 ± 16.4	0.181	0.857
Mitral E/A	0.97 ± 0.4	1.02 ± 0.3	-0.807	0.421
Deceleration time	0.23 ± 0.05	0.22 ± 0.04	1.510	0.133
Septal mitral e	7.84 ± 1.8	8.58 ± 2.3	-2.023	0.044
Septal mitral a	9.13 ± 2.0	9.24 ± 1.8	-0.355	0.723
Mitral E/e	8.56 ± 2.2	8.43 ± 1.8	0.390	0.697

Correlation of CIMT with parameters of LV systolic and diastolic function in hypertensives

There was a positive correlation between CIMT and LV posterior wall (p < 0.001), left ventricular mass index (LVMI) (p = 0.001), and interventricular septal wall thickness (p < 0.001), which was statistically significant, but there was also a positive correlation with left ventricular end-diastolic diameter (LVEDD) (p = 0.112), which was not statistically significant. LVEF (p = 0.625), LV fractional shortening (p = 0.350), and EPSS (p = 0.510) had a positive correlation with CIMT which was not statistically significant. There was a relationship with left atrium (p = 0.001), LV volume index (p < 0.001), and mitral E/A (p = 0.029) but no significant relationship with mitral E/e (p = 0.340) (Table 6).

**Table 6:** Correlation between CIMT and echocardiographic parameters in hypertensive participants. *: P-value <0.05 is statistically significant. CIMT = carotid intima-media thickness; LVEDD = left ventricular end-diastolic diameter; LVSD = left ventricular end-systolic diameter; LVPWD = left ventricular posterior wall thickness in diastole; EPSS = E-point septal separation; EF = ejection fraction; FS = fractional shortening; LVMI = left ventricular mass index; LAVI = left atrial volume index; IVSD = interventricular septal wall thickness in diastole

	Correlation coefficient	P-value
Left atrium	0.248	<0.001*
LVEDD	0.113	0.112
LVESD	0.055	0.438
IVSD	0.359	<0.001*
LVPWD	0.327	<0.001*
EPSS	0.047	0.510
LV EF	0.035	0.625
LV FS	0.066	0.350
LV mass	0.337	<0.001*
LVMI	0.323	<0.001*
LA volume	0.340	<0.001*
LAVI	0.298	<0.001*
Mitral E	-0.121	0.089
Mitral A	0.092	0.194
Mitral E/A	-0.155	0.029*
Deceleration time	0.163	0.021*
Septal mitral e	-0.212	0.003*
Septal mitral a	0.056	0.432
Mitral E/e	0.068	0.340

## Discussion

Correlation of clinical parameters with CIMT

In this study of hypertensive participants, there was a positive correlation between age and CIMT (r = 0.329; p < 0.001). The results of other studies have also indicated a positive correlation between age and CIMT. Umeh et al. [29] reported a significant positive correlation between age and CIMT (left CIMT: r = 0.520, right CIMT: r = 0.427; p = 0.000). Likewise, the study of Ibinaiye et al. [8] also indicated that increasing age was strongly correlated with increasing CIMT (r = 0.35; p = 0.000). Similarly, Vaudo et al. [30] also reported a positive correlation with age (r = 0.38; p = 0.001). In hypertensives, increased CIMT can be due to age. Furthermore, hypertension speeds up the aging process of intima [8].

Concerning blood pressure and CIMT, some studies showed only systolic blood pressure correlated with increased CIMT while other studies found both systolic and diastolic blood pressure being correlated with CIMT. In this study, there was a significant positive correlation between systolic blood pressure (p < 0.001) and CIMT but not with diastolic blood pressure (p = 0.224). This is similar to Nand et al. [31] who observed a significant association of CIMT with systolic blood pressure but not with diastolic blood pressure. In contrast to our study, Khutan et al. [32] and Ibinaiye et al. [8] showed that increased systolic and diastolic blood pressure correlated with increased mean CIMT. The results of Ibinaiye et al. [8] for both systolic (r = 0.22; p = 0.007) and diastolic blood pressure (r = 0.21; p = 0.002) both positively correlated to mean CIMT. Additionally, Khutan et al. [32] observed that CIMT had a statistically significant association with high systolic blood pressure and high diastolic blood pressure (p < 0.001).

There are different reasons why this correlation occurred. Some authors have suggested that the status of blood pressure control can contribute to this finding. Hypertension is a major driver of arterial remodeling and atherosclerosis. The observation that systolic blood pressure was only significantly related to CIMT in this study may be due to the impact of age, where the elderly tend to have more isolated systolic hypertension. In this study, there was no significant correlation between CIMT and BMI (r = 0.052; p = 0.465). Oluwagbemiga et al. [33] also did not show a significant correlation between BMI and CIMT(p > 0.05). However, Vaudo et al. [30] found a weak negative correlation between BMI and CIMT (r = -0.23; p = 0.020 ). In contrast, Sorof et al. [34] found a positive correlation between CIMT and BMI (r = 0.43; p = 0.14) but was not statistically significant.

The pattern of LV geometry in hypertensives

LVH is an independent risk factor for CVDs [11]. In this study, the LV geometry was normal in 50.5%, concentric LV remodeling was seen in 34%, concentric LVH in 9%, and eccentric LVH in 6.5%. Findings of normal LV geometry in the index study vary with similar studies conducted where lower values such as 26.1-38.9% [35], 24% [10], 19.7% [36], 18.8% [37], and 11.3% [38] were observed. Though Adebiyi et al. [35] used different LVMI, Isa et al. [10] studied newly diagnosed hypertensives, and Di Bello et al. [36] studied hypertensives not on medications. All these could account for the reduced normal geometry observed in them compared to our study.

Karaye et al. [37] reported normal geometry mainly in those with a lesser duration of hypertension. Saragih et al. [38] observed that those with normal geometry had a lesser duration of hypertension. All of our study participants were on antihypertensive medications which could be responsible for the increased percentage with normal geometry. Furthermore, the duration of hypertension in this study was <10 years in 75.5% of hypertensives. In this study, concentric LV remodelling was 34% compared to 19.4% [37], 3.7% [36], 23.5% [38], and 17.9-30.2% [35] reported in other studies. Additionally, in this study, concentric LVH was 9%, whereas others observed 37% [10], 25.3% [37], 61.7% [36], 57.4% [38], and 13.4-25.6% [35]. Furthermore, eccentric LVH was 6.5% in this study compared to 36.6% [37], 14.9% [36], 7.8% [38], and 17.5-30.4% [35] in other studies. Karaye et al. [37] reported predominantly eccentric LVH with a more female-to-male ratio, whereas other studies reported predominantly concentric LVH [35,36,38]. Di Bello et al. [36] observed predominantly concentric LVH, possibly due to more males compared to this study with 39% male subjects. Likewise, Saragih et al. [38] also observed that concentric LVH was largely seen in the hypertensives, likely due to the diabetics included in the study.

The LVH demonstrated in this study was 15.5%, which is similar to the 20% Hammond et al. [39] observed in the study of 621 hypertensive individuals due to a higher sample size. Interestingly, in the study by Sorof et al. [34], the prevalence of LVH was 41% among hypertensive children not on treatment and with elevated blood pressure which could account for the increase. LVH is a marker of cardiovascular risk among hypertensive individuals [3].

LV geometry of hypertensive patients with abnormal and normal CIMT

In this study, 22.5% of the hypertensives had abnormal CIMT while 77.5% had normal CIMT. This study observed that 84.5% with normal geometry had normal CIMT. This was in contrast with Saragih et al. [38] who reported 24% normal CIMT, though the cut-off used was much lower than in this study. In this study, 85.3% with concentric LV remodeling, 38.9% with concentric LVH, and 38.5% with eccentric LVH had normal CIMT. However, Saragih et al. [38] observed more hypertensives with normal CIMT had concentric LV remodeling (48%). Those with concentric LVH (20%) with normal CIMT were more than eccentric LVH (8%). In this study, 45% of hypertensive participants with normal geometry had abnormal CIMT. However, Saragih et al. [38] observed a contrasting prevalence. In their study, more of those with concentric LVH (86.2%) had abnormal CIMT, with 1.5% of those with normal LV geometry having abnormal CIMT. Their study included diabetics and smokers which would have likely increased both LVH and CIMT.

In our study, the prevalence of CIMT was higher in hypertensive participants with LVH compared with normal or concentric LV remodelling. This is similar to the Khutan et al. [32] study that reported higher CIMT (45.9%) in hypertensive participants with LVH and abnormal CIMT than LVH with normal CIMT (5.1%). This may be due to a sample population of 100 hypertensives between the ages of 30-55 years excluding the elderly [32]. Additionally, Vaudo et al. [30] reported higher CIMT in LVH (0.84 ± 6 0.16 mm) than in normal geometry (0.71 ± 0.16 mm).

The study involved hypertensives not on medications and no elderly hypertensives such as the Khutan et al. study [32]. Interestingly, Sorof et al. [34] studied 32 pediatric never-treated hypertensives with elevated blood pressure and recorded a higher prevalence of LVH with abnormal CIMT compared with normal geometry at 89% compared with 22% with normal CIMT. These studies have observed increased CIMT with LVH though there are variations that may be attributable to the cut-off value for abnormal CIMT, different sample sizes, sample populations, and some untreated hypertensive participants.

The mean LVMI in this study seen in hypertensive participants with abnormal CIMT was 97.84 ± 30.5 and with normal CIMT was 80.75 ± 15.6, which was statistically significant (p < 0.001). Similarly, Khutan et al. [32] also found a significant association (p < 0.001) between LVMI and CIMT, with LVMI and abnormal CIMT at 50.9 ±20, and normal CIMT was 32 ± 9.9, with 54% being overweight or obese. Sorof et al. [34] also studied hypertensive, and those with increased CIMT had higher LVMI compared with normal CIMT (46.8 g/m^2.7^ vs. 31.4 g/m^2.7^) and they were not on antihypertensives. This may be due to the common pathophysiologic pathway of CIMT and LVMI and neurohumoral and atherosclerotic influences. Moreover, LVMI is an independent predictor of cardiovascular morbidity and mortality.

LV systolic function of hypertensives with abnormal and normal CIMT

In this study, there was no statistical difference between CIMT and LV systolic parameters such as EF (p = 0.916), EPSS (p = 0.334), and LVFS (p = 0.565) of hypertensive participants with normal or abnormal CIMT. This study observed that the mean EF with normal CIMT was 70.20 ± 5.9 and the mean EF with abnormal CIMT was 70.31 ± 7.3 (p = 0.916). Saragih et al. [38] similarly observed no significant difference between mean EF with normal CIMT (63.9 ± 7.7) and mean EF with abnormal CIMT 65.8 ± 7.6 (p = 0.168). However, Mostafa et al. [18], who studied individuals with suspected coronary artery disease, showed that increased CIMT was seen in reduced systolic function which was excluded in this study. Chahal et al. [40] reported reduced LVEF in those with carotid plaque compared with only CIMT possibly due to carotid plaque not being observed in this study, which led to no significant difference in systolic function.

LV diastolic function of hypertensive patients with abnormal and normal CIMT

In this study, the grading of diastolic dysfunction and CIMT showed a significant association (χ^2^ = 7.069, p = 0.029). Notably, some diastolic parameters were significantly different among normal and abnormal CIMT, such as LAVI (t = 4.930, p < 0.001) and septal mitral e (t = -2.023, p = 0.044). Meanwhile, others had no statistical significance, such as mitral E/A (t = 0.421, p = 0.421), DT (t = 1.510, p = 0.133), and mitral E/e (t = 0.390, p = 0.697). However, Saragih et al. [38] reported that septal mitral e (p = 0.003), similar to this study, was statistically different while mitral E/e (p = 0.036) which was statistically different was not in our study. Regarding LAVI, Saragih et al. [38] noted no statistical significance (p = 0.487) unlike our study, which could be a result of the reduced sample size. Kota et al. [25] observed that CIMT and mitral E/A were statistically significant (p = 0.001), unlike our study. This could be due to the small sample size, and the study population was patients with diabetes and ischemic stroke. Similarly, Parrinello et al. [41] observed that abnormal CIMT was more with LV diastolic dysfunction though they were hypertensives <55 years old. This could be a result of increased vascular stiffness due to the hemodynamic changes from hypertension which would worsen heart compliance leading to diastolic dysfunction.

Correlation of LV geometry with CIMT

In this study, the relationship of LV geometry to CIMT was evaluated using univariate and multivariate analyses. Regarding LV geometry, the univariate analysis demonstrated a strong relationship between CIMT with the following individual variables: LAD (r = 0.248, p < 0.001), IVSD (r = 0.359, p < 0.001), LVM (r = 0.337, p < 0.001), LVMI (r = 0.323, p < 0.001). However, on multivariate, only IVSD (p = 0.017) maintained a significant relationship. Concerning LV geometry, a strong association was noticed between CIMT and the different grades of geometry (χ^2^ = 31.688, p < 0.001). However, multivariate eccentric LV geometry indicated a relationship (p = 0.005).

Several studies have observed a strong association between LVH and CIMT, which this study has also noted can increase the risk of cardiovascular events and can be a sign of hypertension-mediated end-organ damage. There was a positive correlation between CIMT and LVMI (r = 0.323, p = 0.001) in this study. Similarly, in the study by Khutan et al. [32], CIMT had a significant association with LVMI (p < 0.001). CIMT and LVMI have similar pathophysiology which is the hypertrophic response and could be responsible for their association, especially in hypertensives. In the study by Sorof et al. [34], there was a relationship between CIMT and LVMI (r = 0.58, p < 0.01). There was a positive correlation between CIMT and LV posterior wall (p < 0.001) and interventricular septal wall thickness (p < 0.001) which was statistically significant in this study. Similarly, Sorof et al. [34] showed that CIMT was positively correlated with the interventricular septal thickness (r = 0.58; p < 0.001) and posterior wall thickness (r = 0.54; p < 0.001) which were statistically significant. These parameters which are used to determine LV geometry also showed a significant association with CIMT which further reinforces the effect of LV geometry and CIMT.

Correlation of LV function and CIMT

Correlation of univariate and multivariate CIMT with different parameters of systolic function did not show any correlation: LVEF (r = 0.035, p = 0.625), LV fractional shortening (r = 0.066, p = 0.350), and EPSS (r = 0.047, p = 0.510). However, Yarynkina et al. [42] studied 77 hypertensive and CIMT with EF and observed a positive significant correlation (r = 0.20, p = 0.05). This is in contrast to the study done by Rafieian-Kopaei et al. [43] who noted a negative correlation between CIMT and EF which was significant (r = -0.353, p = 0.005). This was possibly due to the sample population who were hypertensives and diabetic with end-stage renal disease. Rafieian-Kopaei et al. [43] also observed accelerated atherosclerosis in diabetics with renal impairment.

Correlation of univariate and multivariate CIMT with different parameters of diastolic function showed varying correlation: LAVI (r = 0.298, p < 0.001), mitral E/A (r = -0.121, p = 0.089), DT (r = 0.163, p = 0.021), mitral E/e (r = 0.068, p = 0.340), septal mitral e (r = -0.212, p = 0.003). However, on multivariate analysis, only DT (p = 0.025) maintained a significant relationship. Systemic hypertension is associated with diastolic dysfunction and CIMT. This study showed a significant correlation between the left atrium and CIMT (r = 0.248, p = 0.001). Similarly, Yarynkina et al. [42] also observed a positive correlation between the left atrium and CIMT (r = 0.30, p = 0.01). This is possibly due to systemic hypertension, and in some studies, left atrium diameter can predict increased CIMT.

Limitations

In this study, CIMT measurements were done manually. Automated edge detection is preferred and more accurate as the manual method is operator-dependent. Additionally, as the study was hospital-based in a single tertiary hospital, the results might not be generalizable to all hypertensives in society.

## Conclusions

This study described CIMT assessment and correlation with echocardiographic LV geometry and function in hypertensive patients. Hypertensives are at risk of hypertension-mediated organ damage which includes LVH and abnormal CIMT. Abnormal CIMT has been shown to be higher in hypertensives with concentric and eccentric LVH, which can further increase their risk of cardiovascular events. LVH, which is an independent risk factor for cardiovascular events, has been shown to be associated with abnormal CIMT which further increases the risk of CVDs.
